# National surveillance of hookworm disease in China: A population study

**DOI:** 10.1371/journal.pntd.0010405

**Published:** 2022-06-09

**Authors:** Hui-Hui Zhu, Ji-Lei Huang, Ying-Dan Chen, Chang-Hai Zhou, Ting-Jun Zhu, Men-Bao Qian, Mi-Zhen Zhang, Shi-Zhu Li, Xiao-Nong Zhou

**Affiliations:** 1 National Institute of Parasitic Diseases, Chinese Center for Disease Control and Prevention (Chinese Center for Tropical Diseases Research), NHC Key Laboratory of Parasite and Vector Biology, WHO Collaborating Centre for Tropical Diseases, National Center for International Research on Tropical Diseases, Shanghai, China; 2 School of Global Health, Chinese Center for Tropical Diseases Research-Shanghai Jiao Tong University School of Medicine, Shanghai, China; Ben-Gurion University of the Negev, ISRAEL

## Abstract

**Background:**

Hookworm disease is endemic in China and is widespread globally. The disease burden to humans is great.

**Methods:**

The study described the national surveillance of hookworm implemented in 31 provinces/autonomous regions/municipalities (P/A/Ms) of China in 2019. Each P/A/M determined the number and location of surveillance spots (counties). A unified sampling method was employed, and at least 1000 subjects were investigated in each surveillance spot. The modified Kato-Katz thick smear method was employed for stool examination. Fifty samples positive with hookworm eggs were cultured in each surveillance spot to discriminate species between *A*. *duodenale* and *N*. *americanus*. Twenty-five soil samples were collected from each surveillance spot and examined for hookworm larva. The 2019 surveillance results were analyzed and compared with that of 2016–2018.

**Results:**

A total of 424766 subjects were investigated in 31 P/A/Ms of China in 2019, and the overall hookworm infection rate was 0.85% (3580/424766). The weighted infection and standard infection rates were 0.66% (4288357/648063870) and 0.67% (4343844/648063870), respectively. Sichuan province had the highest standard infection rate (4.75%) in 2019, followed by Chongqing (2.54%) and Hainan (2.44%). The standard infection rates of other P/A/Ms were all below 1%, with no hookworm detected in 15 P/A/Ms. The standard hookworm infection rate in the males and the females were 0.61% (2021216/330728900) and 0.71% (2267141/317334970), respectively, with a significant difference between different genders ($\chi^{2}$ = 17.23, *P<*0.0001). The highest standard hookworm infection rate (1.97%) was among age ≥ 60 years, followed by 45~59 years (0.77%), 15~44 years (0.37%), and 7~14 years (0.20%). The lowest standard infection rate was among the 0~6 years age group (0.12%). A significant difference was observed among different age groups ($\chi^{2}$ = 2 305.17, *P<*0.0001). The constitute ratio for *N*. *americanus*, *A*. *duodenale*, and coinfection was 78.70% (1341/1704), 2.03% (346/1704), and 1.00% (17/1704), respectively. The detection rate of hookworm larva from soil was 3.45% (71/2056).

**Conclusion:**

The national surveillance showed that the hookworm infection rate has been decreasing annually from 2016 to 2019, and it is now below 1%. China has made significant progress in controlling hookworm. The national surveillance system is an important way to understand the endemic status and provide important information in this process and thus needs to be continually optimized.

## Author summary

Hookworm disease is endemic in China. The national surveillance system on important parasitic diseases including hookworm infection has been established in China. Stool samples were collected from participants, and the Kato-Katz method was applied to detect helminth eggs while samples with hookworm eggs were further cultured to differentiate the species of the parasite. Additionally, soil samples were collected and examined for hookworm larva. In 2019, the overall infection rate of hookworm in China was 0.85% (3580/424766). High prevalence was demonstrated in Western and Southern China, including Sichuan (4.75%), Chongqing (2.54%) and Hainan (2.44%). The prevalence was high in the females (0.71%) than in the males (0.61%), while it was high in older population especially those age over 60 years. *N*. *americanus* dominated the hookworm species. The prevalence of hookworm in soil was 3.45%. Overally, hookworm infection decreased to a low level in China. However, there still exist high endemic areas. Thus, intervention needs to be applied in the high endemic areas and elder population.

## Background

Hookworm (*Necator americanus* and *Ancylostoma duodenale*) is a kind of soil-transmitted helminth (STH) endemic worldwide, causing harm to both children and adults [1–3]. It mainly causes iron deficiency anemia and malnutrition [4–7], leading to more serious consequences such as lethargy, impaired physical and cognitive development, and poor pregnancy outcomes [2,8]. Other consequences, such as obscure overt gastrointestinal bleeding [9] and metabolic disorder in obese individuals [10], were also reported. Of the two species, *N*. *americanus* is the most widespread hookworm, distributed in sub-Saharan Africa, the Americas, and Asia, whereas *A*. *duodenale* is focal. Globally, about 472 million persons were infected with hookworm [11,12], causing the disease burden of 4 million DALYs, with productivity losses of up to US$ 139 billion annually [8,13].

In the People’s Republic of China, there had been three national surveys on parasitic diseases, including soil-transmitted helminthiasis. The first, second, and third national surveys were carried out in 1988–1992, 2001–2004, and 2014–2015, respectively. Hookworm disease prevalence at the first national survey was 17.2%, corresponding to 194 million infected persons in the whole country, which was lower than ascariasis (47.0%, 531 million) and trichuriasis (18.8%, 212 million) [14–16]. Further, hookworm disease prevalence decreased to 6.12% in the second national survey, with 39.30 million estimated number of infection, which was lower than that of ascariasis (12.72%, 85.93 million) yet higher than trichuriasis (4.63%, 29.09 million) [16–18]. However, in the latest national survey, which is the third national survey, the prevalence of hookworm disease was found to be 2.62%, corresponding to 16.97 million infected persons, which was the highest among that of ascariasis (1.36%, 8.83 million) and trichuriasis (1.02%, 6.60 million) [16,19].

In 2020, the road map for neglected tropical diseases 2021–2030 was issued by WHO, and targets were set for the number of countries validated for elimination of soil-transmitted helminthiases as a public health problem. This was defined as <2% proportion of STH infections of moderate and heavy intensity due to *Ascaris lumbricoides*, *Trichuris trichuria*, *Necator americanus*, and *Ancylostoma duodenale* in 70 countries by 2025, and 96 by 2030 [20]. Moreover, the 2030 targets for soil-transmitted helminthiases control programs were newly issued, explaining the indicators to be achieved by 2030 [21]. In China, following the accomplishment of the “National Control Program for Echinococcosis and Other Key Parasitic Diseases (2020–2025)”, the new control program from 2021 to 2025 is now under discussion, and the goal will be set according to the WHO targets for control and elimination of STH infections.

Remarkable achievements have been made in controlling hookworm disease, with the apparent decline in the infection rate and the number of infected persons. Under the current condition, the elimination of hookworm infections has become the most important task in STH elimination, and still, lots of work is necessary. In 2019, the national control pilots for STH infections were established in 12 counties of 9 P/A/Ms endemic with STH. They will last for a minimum of 5 years; new control strategies will be explored to carry out these control pilots. Thus, the national surveillance system is ideal for understanding endemic regularity and providing data support for implementing control activities and evaluating control efforts.

Therefore, after the third national survey, a national surveillance system for soil-transmitted helminthiasis was established in 2016 [22,23] and consecutively implemented for 4 years. About 300 thousand residents were investigated each year from 2016 to 2018, and the infection rates of hookworm were 1.35%, 1.00%, 0.89%, respectively [23–25]. The latest national surveillance was carried out in 2019, and the results were reported in this article. Moreover, the endemic characteristics of hookworm disease in China were analyzed and the results were compared with that of 2016–2018.

## Methods

### Ethics statement

This article was based on an analysis of routine surveillance data from the National Institute of Parasitic Diseases (NIPD) at Chinese Center for Disease Control and Prevention (China CDC), and got the approval from the Institutional Ethical Review Committee of NIPD with the document No.2021006. The project leaders and staff led the review, analysis, and interpretation of the data. No personal information was disclosed. Written informed consent for publication was obtained from all participants.

### Surveillance spots

This national surveillance was implemented in 31 P/A/Ms of China in 2019. Considering spots’ representativeness and work feasibility, each P/A/M determined the number and location of surveillance spots (counties), such that 10%-15% of counties, including the high, middle, and low endemic spots, were covered each year. All the surveillance spots employed the unified sampling method proposed by China CDC. Each spot (county) was divided into five parts according to the geographical orientation (East, West, North, South, and Middle) within the selected county. Five towns were selected from each geographical orientation of the county, then one village from each town was selected so that a total of five villages were selected from each spot (county). No less than 200 subjects were chosen from each selected village by cluster random sampling, so that a total of more than 1000 subjects were investigated in each surveillance spot (county).

### Feces collection and examination

County-level staff distributed feces containers in each village, and feces samples (>30 g) were collected the next day, the feces were stored in room temperature and slides were made of the feces (two slides for each sample) within 2 days using Kato-Katz’s method [26]. The slides were read by county- or provicial-level professional staff and then stored in 0–4°C for future re-check. Fifty samples with hookworm eggs were cultured for each surveillance spot to discriminate two species between *A*. *duodenale* and *N*. *americanus*. All the positive samples were cultured if less than 50 positive samples were found per spot.

About 0.5 g of feces sample was smeared on the center of a banned filter paper, and the paper was put into a tube with water, maintaining the water level in the bottom part of the filter paper but not reach the stool sample. The tube was cultured in a moist atmosphere at 31°C for 4 days or 26~30°C for 6~8 days. The filter paper with the sample was then dipped into 45°C water in a beaker for one hour to allow hookworm larva to swim into the water. The water was kept still in the beaker so that the larva can sink to the bottom. After another hour, the supernatant was poured out, keeping 0.5 ml water with the larva at the bottom. The remaining water was dipped on a slide and examined under the anatomical microscope to identify the hookworm lava according to morphology [27].

### Soil examination

Five households were selected randomly from each village, and surface soil was collected from fields or vegetable yards of each household for soil examination. About 350 g soil was crumbled before putting them on a three-layer cotton paper, and both were put into a mesh screen. The cotton paper was sunk into 5% saline water through the mesh screen and kept at 45°C for 1.5 h for the hookworm larva to swim into the water. Then, the saline water was collected in a beaker and kept for 15 minutes for natural sedimentation. The supernatant was discarded, leaving 50 ml or less saline water. The saline water was then put into a culture dish and examined under an anatomical microscope to see if there were hookworm larvae. The saline water with hookworm larva was examined again under a microscope for identification of hookworm species.

### Data report and analyses

Data were reported to the "Information Management System for Parasitic Diseases" by each surveillance spot right after surveillance. Excel 2010 and SAS 9.3 (version 70068130) were employed for data analysis. The variables including infection rate, standard infection rate (infection rate adjusted by the demographic distributions of the overall population in China), weighted infection rate (infection rate adjusted by the demographic distributions of each P/A/M) of hookworm in different P/A/Ms, sex, age group, occupation, and educational level were analyzed. For calculation of standard infection rate, the demographic distribution data, which includes the age and gender distribution of the overall population in China, were employed. Specifically speaking, the age group was defined as 0~5, 5~10, 10~15… 80~85, 85 yeas and above with 5 years’ gap, and the population of each gender and age group in national surveillance was calculated and defined as “count”, moreover, the population of each gender and age group in the overall population in China was also calculated and defined as “pop”. The standard weight was defined as pop/count in each gender and age group, and the standard infection rate was calculated according to the standard weight. For calculation of weighted infection rate, the demographic distribution data, which includes the age and gender distribution of each P/A/M in China, were employed. Specifically speaking, the age group was also defined with 5 years’ gap, and the weighted weight in each P/A/M was calculated as aforementioned, except that the demographic distribution data in each P/A/M were used. The weighted infection rates in each P/A/M and in the country were calculated according to the weighted weight. In addition, the constituent ratio of infection intensity and hookworm species were calculated. The mild, moderate, and heavy hookworm infections were defined as EPG(eggs per gram)<2000, 2000 ≤EPG<4000, and EPG ≥4000, respectively. The Chi-square test was used to compare infection rates, and the Cochran-Armitage trend test was used to analyze the changing trend of infection rates, and a significant difference was set as α<0.05.

### Quality control

The training for provincial disease control agencies on surveillance manual/ examination method/ data entry was carried out annually by NIPD at China CDC. The provincial-level agency took the responsibility to train staff at the county level. In addition, 10% of the positive stool samples and 5% of the negative samples were reexamined by the provincial agency each year; the NIPD at China CDC’s staff implemented reexamination of samples randomly. Surveillance data were checked at the municipal and provincial levels consecutively before reporting to the national level. The NIPD at China CDC’ staff rechecked the reported data and identified irregularities to correct all problems before data analyses.

## Results

### Distribution of surveillance spots

**Fig 1:**
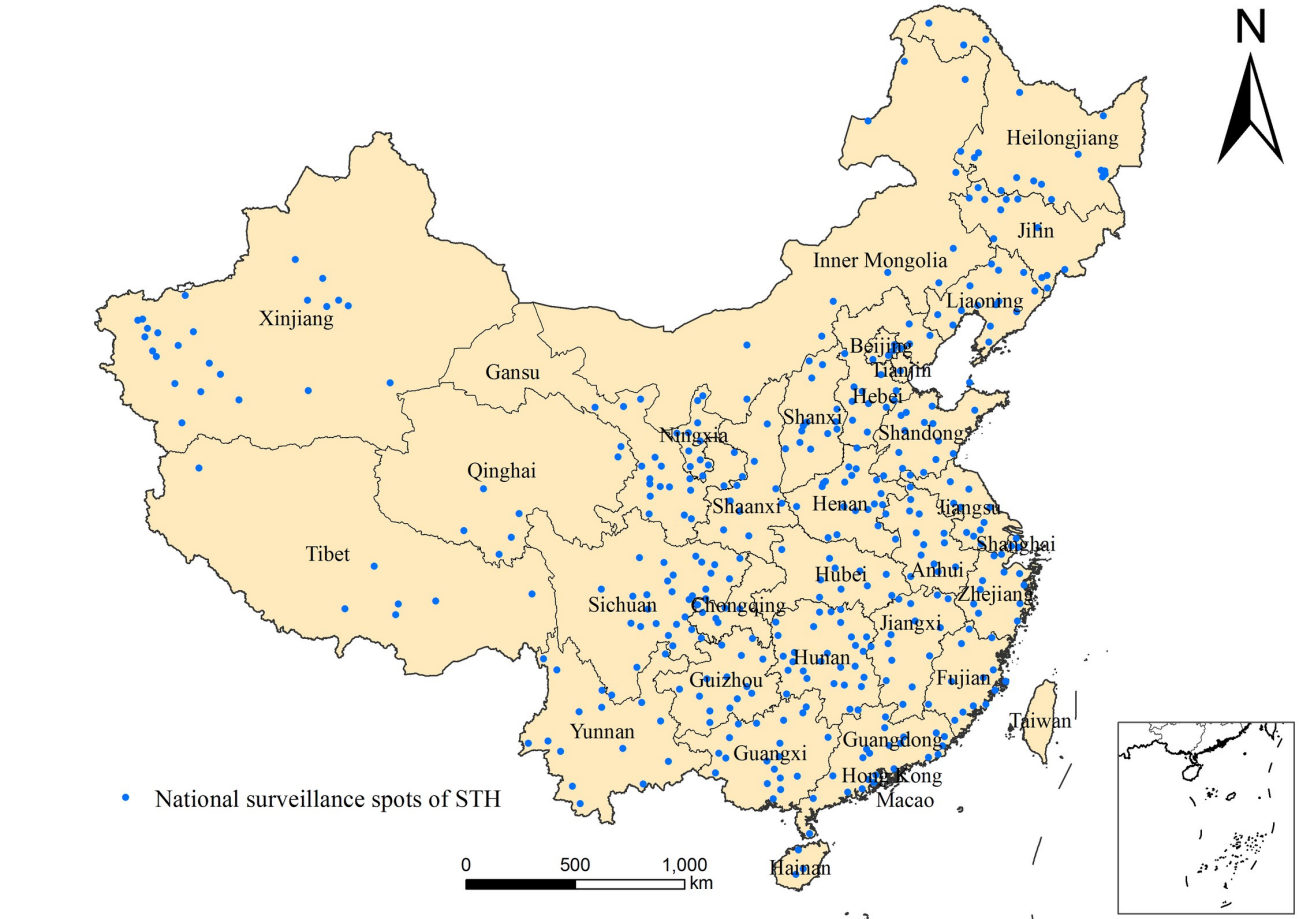
Distribution of national surveillance spots of STH in 2019. (https://www.webmap.cn/mapDataAction.do?method=forw&resType=5&storeId=2&storeName=%E5%9B%BD%E5%AE%B6%E5%9F%BA%E7%A1%80%E5%9C%B0%E7%90%86%E4%BF%A1%E6%81%AF%E4%B8%AD%E5%BF%83&fileId=BA420C422A254198BAA5ABAB9CAAFBC1).

A total of 414 surveillance spots (counties) from 31 P/A/Ms were surveyed in 2019, and the distribution of surveillance spots is shown in Fig 1.

### Results of quality control

The quality control random examination was carried out in 2019, and a total of 2100 slides from Jilin, Jiangxi, Hunan, Guangxi, and Heilongjiang were examined. The consistency rate was 99.81% (2096/2100), showing a high fecal examination quality.

### Overall prevalence and P/A/M distribution

**Fig 2:**
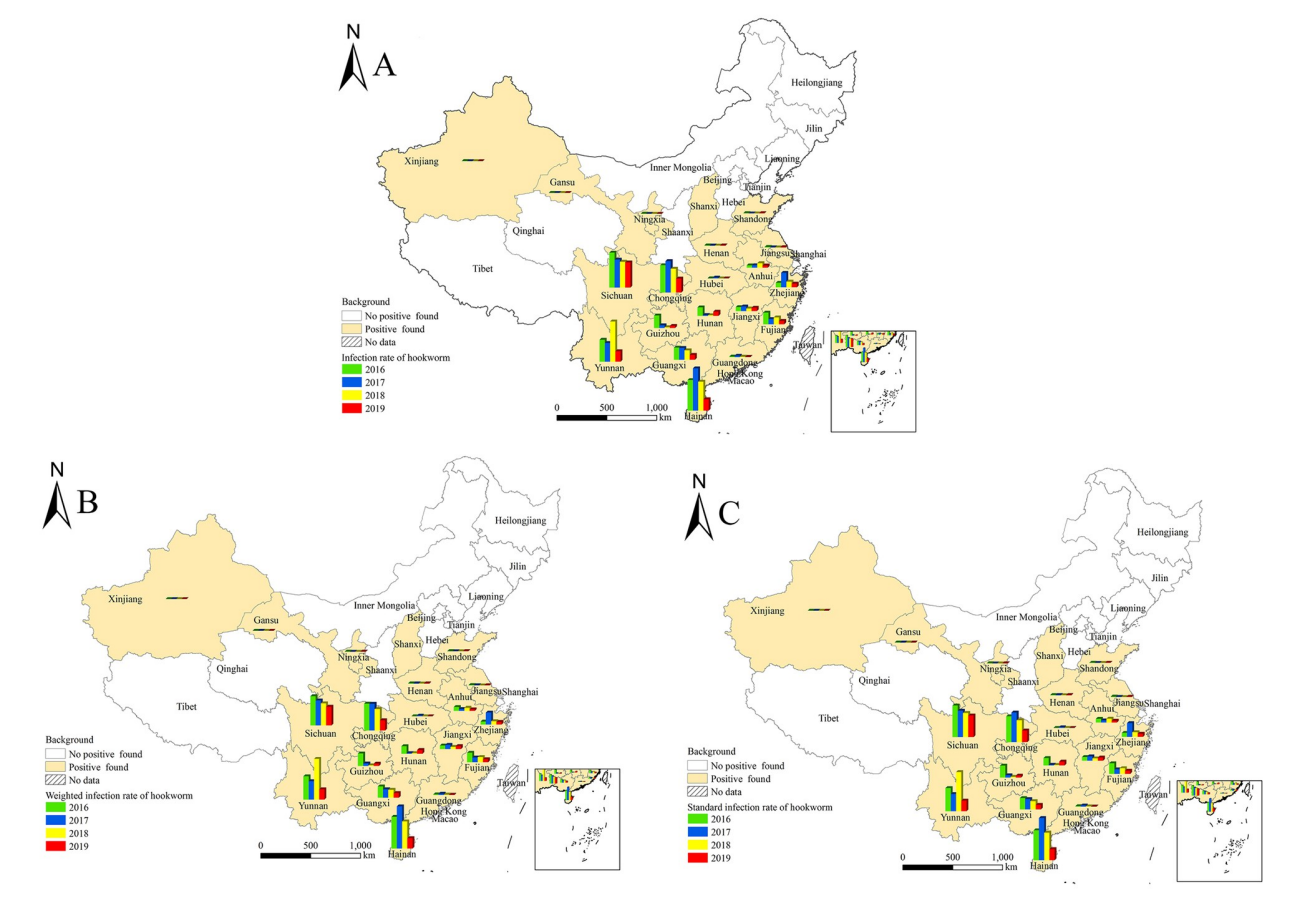
Distribution of hookworm infections in different P/A/Ms from 2016 to 2019 according to the national surveillance in China. A. Infection rate; B. Weighted infection rate; C. Standard infection rate. (https://www.webmap.cn/mapDataAction.do?method=forw&resType=5&storeId=2&storeName=%E5%9B%BD%E5%AE%B6%E5%9F%BA%E7%A1%80%E5%9C%B0%E7%90%86%E4%BF%A1%E6%81%AF%E4%B8%AD%E5%BF%83&fileId=BA420C422A254198BAA5ABAB9CAAFBC1).

Overall, 424766 subjects were investigated in 31 P/A/Ms of China in 2019. The overall hookworm infection rate was 0.85% (3580/424766), and the weighted infection rate and standard infection rate were 0.66% (4288357/648063870) and 0.67% (4343844/648063870), respectively. The highest standard infection rate was in Sichuan province in 2019, which was 4.75%, followed by 2.54% in Chongqing, 2.44% in Hainan and 2.34% in Yunnan. The standard infection rate of other P/A/Ms was all below 1%, with no hookworm detected in 15 P/A/Ms (Table 1). From 2016 to 2019, hookworm was mainly distributed in the southwest part of China. The first four highest standard infection rate were all distributed in Sichuan, Chongqing, Hainan, and Yunnan with different sequences, and no hookworm was found in 10 P/A/Ms namely Beijing, Tianjin, Hebei, Inner Mongolia, Liaoning, Jilin, Heilongjiang, Shanghai, Shaanxi, and Qinghai for 4 successive years [23–25] (Fig 2).

**Table 1:** Distribution of hookworm infections in different P/A/Ms in 2019 according to the national surveillance.

P/A/Ms	Raw data			Weighted data			Standard data		
	No. of examination	No. of infections	Infection rate (%)	Weighted No. of examination	Weighted No. of infections	Weighted infection rate (95% CI, %)	Standard No. of examination	Standard No. of infections	Standard infection rate (95% CI, %)
Beijing	3008	0	0.00	2650620	.	.(.-.)	4225093	.	.(.-.)
Tianjin	2028	0	0.00	2560530	.	.(.-.)	3160088	.	.(.-.)
Hebei	15239	0	0.00	40038920	.	.(.-.)	23185136	.	.(.-.)
Shanxi	14189	0	0.00	18275040	.	.(.-.)	21604718	.	.(.-.)
Inner Mongolia	10185	0	0.00	10327120	.	.(.-.)	15022794	.	.(.-.)
Liaoning	14085	0	0.00	16373900	.	.(.-.)	21512925	.	.(.-.)
Jilin	12000	0	0.00	12089980	.	.(.-.)	17898491	.	.(.-.)
Heilongjiang	19082	0	0.00	15494330	.	.(.-.)	29935961	.	.(.-.)
Shanghai	3090	0	0.00	2412080	.	.(.-.)	4597193	.	.(.-.)
Jiangsu	11538	11	0.10	30930970	16248	0.05(0.02–0.09)	15135371	10703	0.07(0.03–0.11)
Zhejiang	11538	100	0.87	20926620	117813	0.56(0.43–0.70)	15565036	110206	0.71(0.56–0.85)
Anhui	14982	78	0.52	30748740	98479	0.32(0.23–0.41)	20154826	79611	0.39(0.30–0.49)
Fujian	12686	100	0.79	15071350	97042	0.64(0.46–0.83)	18066582	122062	0.68(0.52–0.83)
Jiangxi	14203	115	0.81	24605730	157885	0.64(0.49–0.79)	21943881	151074	0.69(0.55–0.83)
Shandong	14537	10	0.07	48023490	25919	0.05(0.02–0.09)	21195261	11109	0.05(0.02–0.09)
Henan	21660	18	0.08	57822390	29912	0.05(0.03–0.08)	32627973	17345	0.05(0.03–0.08)
Hubei	10168	0	0.00	27247410	.	.(.-.)	14342525	.	.(.-.)
Hunan	28092	278	0.99	35411410	293590	0.83(0.71–0.95)	40890154	337761	0.83(0.71–0.94)
Guangdong	22879	13	0.06	32212670	19685	0.06(0.02–0.10)	38527645	18519	0.05(0.01–0.08)
Guangxi	20370	233	1.14	26357750	265036	1.01(0.86–1.15)	30739074	305241	0.99(0.85–1.14)
Hainan	3205	86	2.68	4282370	97075	2.27(1.70–2.84)	5005796	122278	2.44(1.87–3.02)
Chongqing	6149	202	3.29	12346140	271667	2.20(1.85–2.55)	8480104	215150	2.54(2.16–2.91)
Sichuan	31292	1849	5.91	51194260	2082128	4.07(3.83–4.30)	43255600	2055596	4.75(4.51–4.99)
Guizhou	14151	72	0.51	22482390	110525	0.49(0.37–0.61)	21868328	95447	0.44(0.32–0.55)
Yunnan	16693	414	2.48	29732710	660160	2.22(1.98–2.46)	27000729	632223	2.34(2.09–2.59)
Tibet	6441	0	0.00	2058680	.	.(.-.)	11463091	.	.(.-.)
Shaanxi	9053	0	0.00	20446730	.	.(.-.)	14410387	.	.(.-.)
Gansu	18705	0	0.00	17600290	.	.(.-.)	29709796	.	.(.-.)
Qinghai	7043	0	0.00	3026970	.	.(.-.)	13501837	.	.(.-.)
Ningxia	12017	1	0.01	3255750	680	0.02(0.00–0.06)	20224463	4031	0.02(0.00–0.06)
Xinjiang	24458	0	0.00	12056530	.	.(.-.)	42813010	.	.(.-.)
Total	424766	3580	0.84	648063870	4343844	0.67(0.64–0.70)	648063870	4288357	0.66(0.64–0.69)

### Area distribution

**Fig 3:**
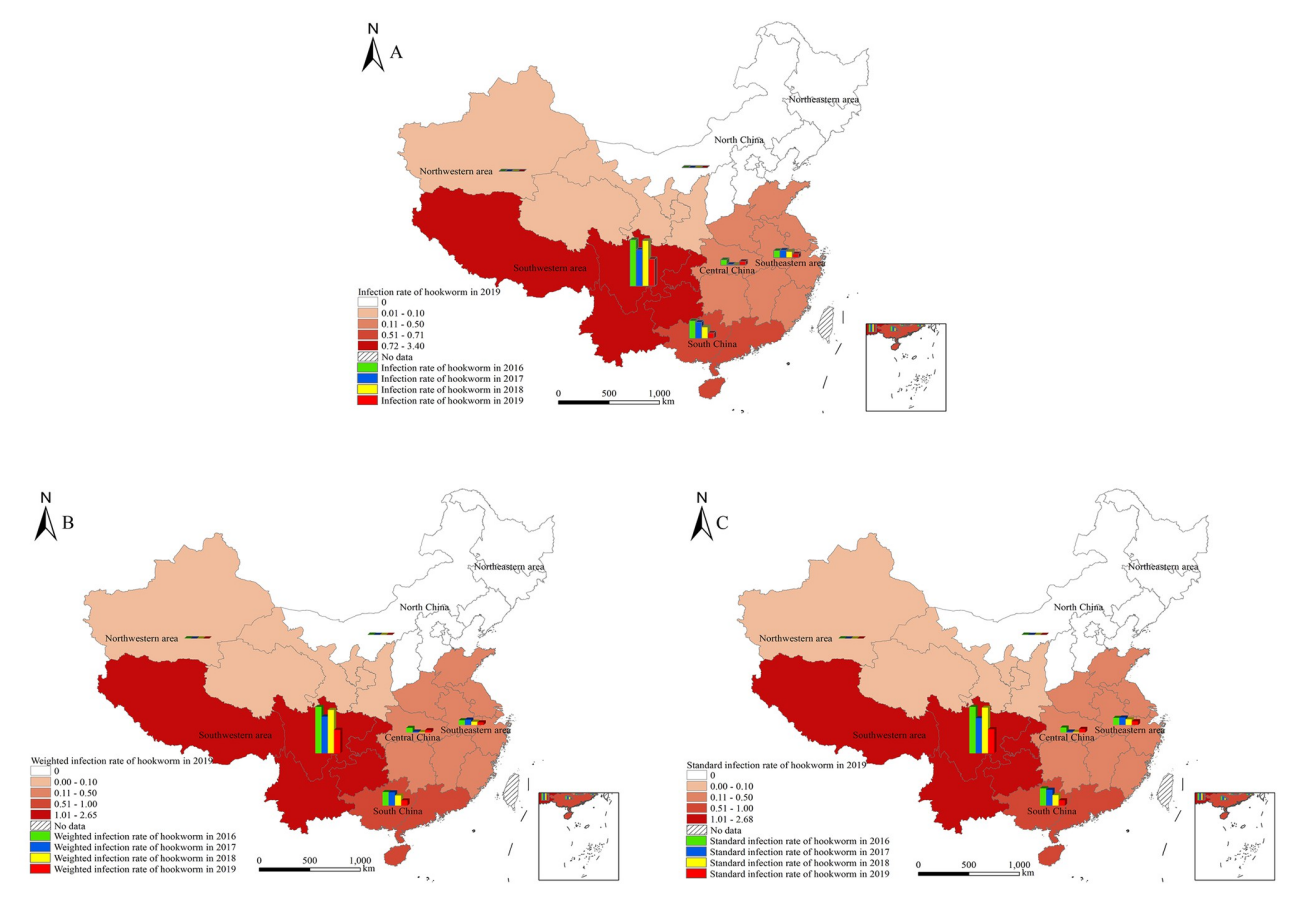
Spatial distribution of hookworm infections from 2016 to 2019 according to the national surveillance in China. A. Infection rate; B. Weighted infection rate; C. Standard infection rate. (https://www.webmap.cn/mapDataAction.do?method=forw&resType=5&storeId=2&storeName=%E5%9B%BD%E5%AE%B6%E5%9F%BA%E7%A1%80%E5%9C%B0%E7%90%86%E4%BF%A1%E6%81%AF%E4%B8%AD%E5%BF%83&fileId=BA420C422A254198BAA5ABAB9CAAFBC1).

According to the area distribution in 2019, the standard infection rate was highest in the Southwestern area, which was 2.68%, followed by South China and Southeastern China. The infection rate was 0.60% and 0.42%, respectively. No infection was found in the Northeastern area, Northwestern area, and North China (Table 2). Similar distribution features were discovered in hookworm standard infection rate from 2016 to 2019, with an inclining trend annually and the highest infection in the southern part (Tibet not included from 2016 to 2018). No infection was found in the Northeastern area, and few or no infection was observed in the Northwestern area and North China (Fig 3).

**Table 2:** Distribution of hookworm infections in different areas in 2019 according to the national surveillance.

Areas	Raw data			Weighted data			Standard data		
	No. of examination	No. of infections	Infection rate (%)	Weighted No. of examination	Weighted No. of infections	Weighted infection rate (95% CI, %)	Standard No. of examination	Standard No. of infections	Standard infection rate (95% CI, %)
Northeastern area	45167	0	0	43958210	.	.(.-.)	69347377	.	.(.-.)
Southeastern area	82574	414	0.5	172718980	513386	0.30(0.26–0.33)	116658152	484765	0.42(0.37–0.46)
North China	44649	0	0	73852230	.	.(.-.)	67197828	.	.(.-.)
South China	46454	332	0.71	62852790	381796	0.61(0.53–0.68)	74272515	446038	0.60(0.53–0.67)
Central China	59920	296	0.49	120481210	323502	0.27(0.23–0.31)	87860652	355106	0.40(0.35–0.46)
Northwestern area	71276	1	0	56386270	680.14	0.00(0.00–0.00)	120659494	4031	0.00(0.00–0.01)
Southwestern area	74726	2537	3.4	117814180	3124481	2.65(2.53–2.78)	112067852	2998417	2.68(2.56–2.79)
Total	424766	3580	0.84	648063870	4343844	0.67(0.64–0.70)	648063870	4288357	0.66(0.64–0.69)

### Gender and age distribution

**Fig 4:**
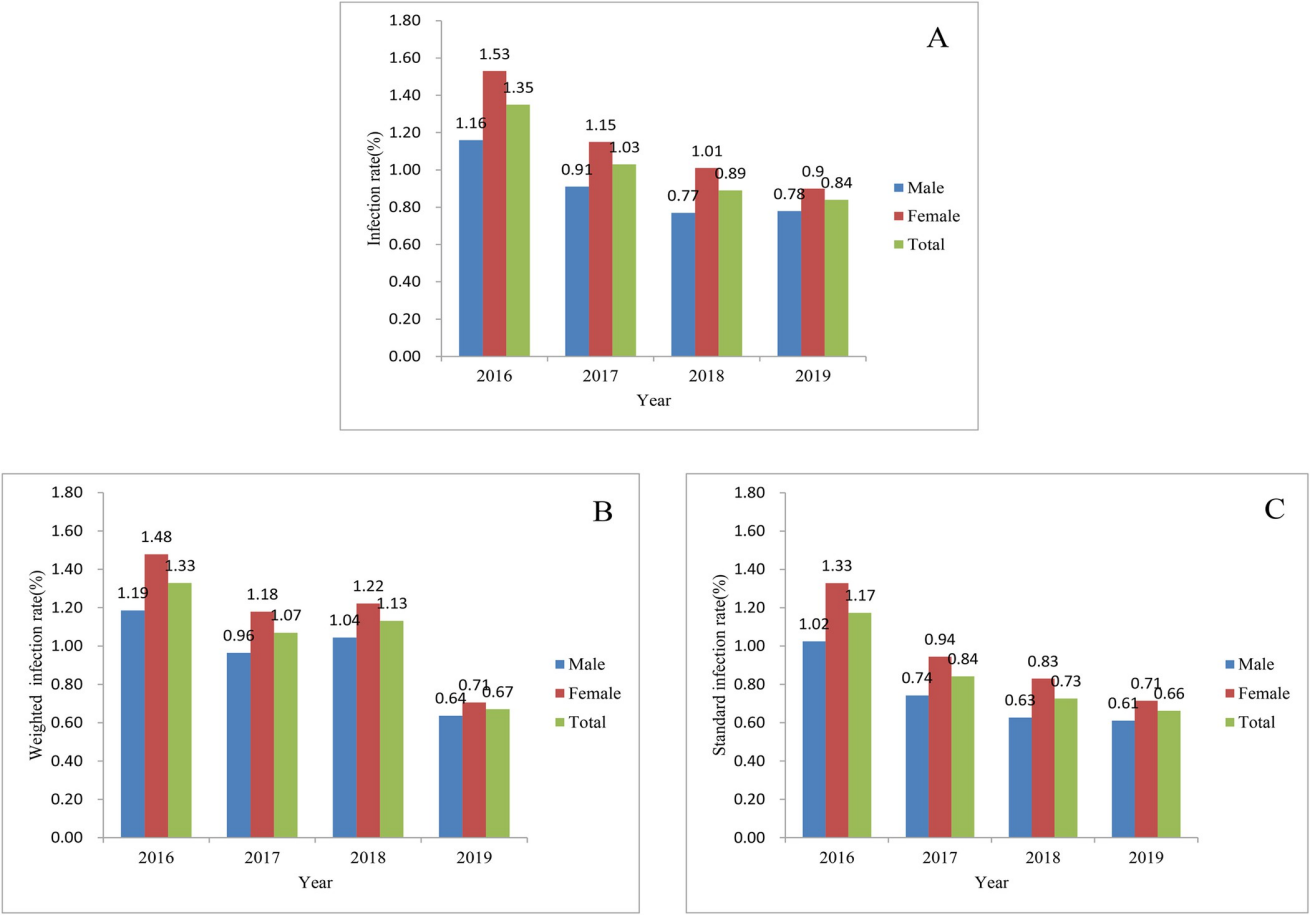
Gender distribution of hookworm infections from 2016 to 2019 according to the national surveillance. A. Infection rate; B. Weighted infection rate; C. Standard infection rate.

The infection rate of hookworm in males and females was 0.78% (1614/207188) and 0.90% (1966/217578), respectively, with the standard infection rate of 0.61% (2021216/330728900) and 0.71% (2267141/317334970), respectively. According to the standard infection rate, there was a significant difference between different genders ($\chi^{2}$ = 17.23, *P*<0.0001). The standard infection rates in females were all higher than that of the males from 2016 to 2019, and they were all significantly different (*P*<0.0001) (Fig 4). In 2019, the highest standard infection rate of hookworm was in 60 years and older age groups, which was1.97%, followed by 45~59 years (0.77%), 15~44 years (0.37%), and 7~14 years (0.20%). The lowest standard infection rate was among the 0~6 years age group (0.12%) (Table 3). A significant difference was observed among different age groups ($\chi^{2}$ = 2 305.17, *P*<0.0001).

**Table 3:** Infection rate of hookworm among different age groups in 2019 according to the national surveillance.

Age groups	Raw data			Weighted data			Standard data		
	No. of examination	No. of infections	Infection rate (%)	Weighted No. of examination	Weighted No. of infections	Weighted infection rate (95% CI, %)	Standard No. of examination	Standard No. of infections	Standard infection rate (95% CI, %)
0~6 years	38501	51	0.13	61649014	100302	0.16(0.11–0.22)	62180186	74811	0.12(0.08–0.16)
15~44 years	115231	430	0.37	285958820	1247764	0.44(0.39–0.48)	285958820	1059541	0.37(0.33–0.41)
45~59 years	104218	824	0.79	134676010	981693	0.73(0.68–0.78)	134676010	1034318	0.77(0.71–0.82)
60 years and above	108260	2163	2.00	100879580	1846418	1.83(1.75–1.91)	100879580	1990534	1.97(1.89–2.06)
7~14 years	58556	112	0.19	64900446	167667	0.26(0.21–0.31)	64369274	129153	0.20(0.16–0.24)
Total	424766	3580	0.84	648063870	4343844	0.67(0.64–0.70)	648063870	4288357	0.66(0.64–0.69)

**Fig 5:**
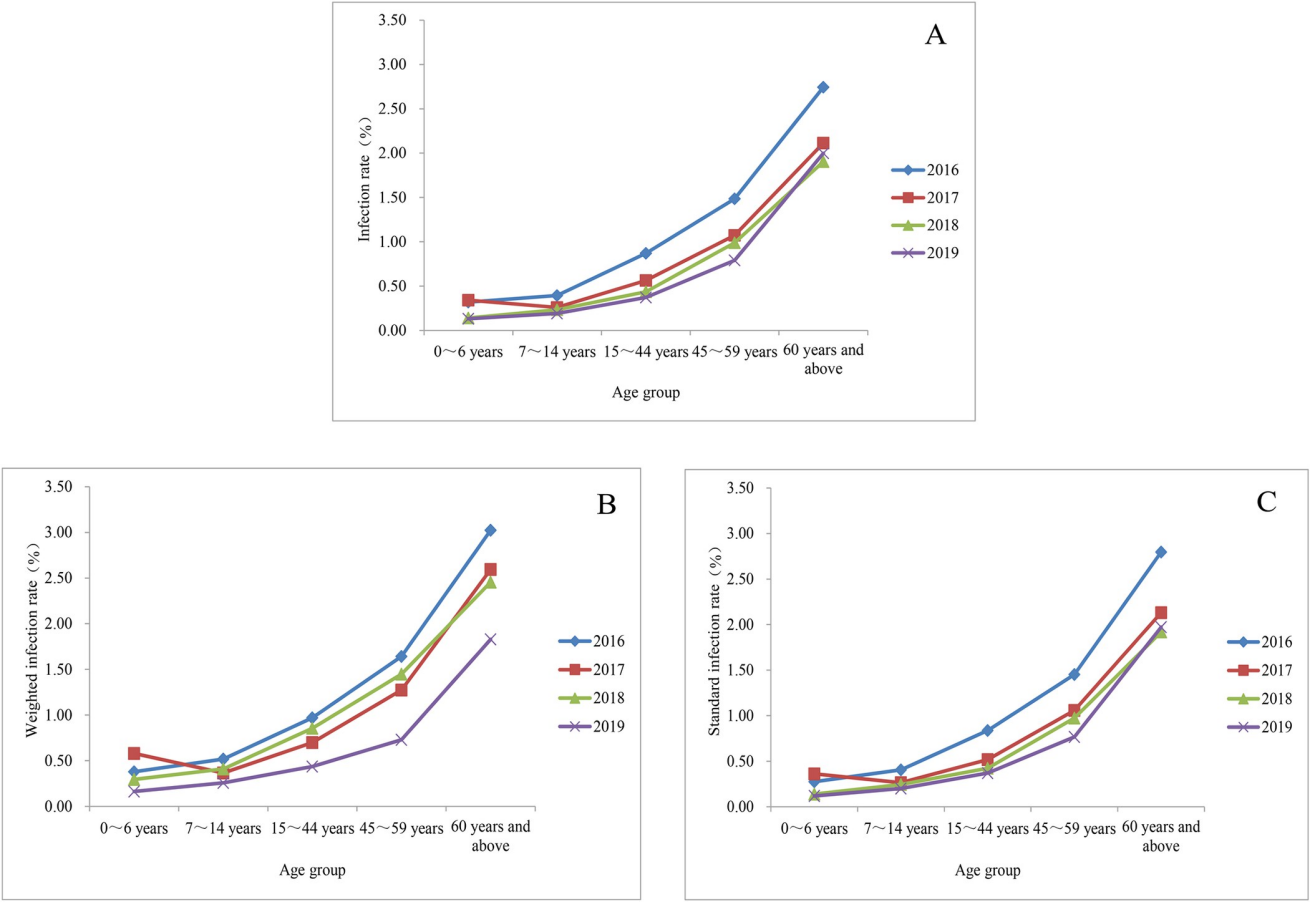
Age distribution of hookworm infections from 2016 to 2019 according to the national surveillance. A. Infection rate; B. Weighted infection rate; C. Standard infection rate.

The age distribution patterns of infections rates from 2016 to 2018 were similar to that of 2019, and a significant difference was observed among different age groups in these 3 years (*P*<0.0001) (Fig 5). The Cochran-Armitage trend test showed an increasing trend in infection rate with increased age in all four years’ national surveillance (*P*<0.0001).

### Educational level and occupation distribution

**Fig 6:**
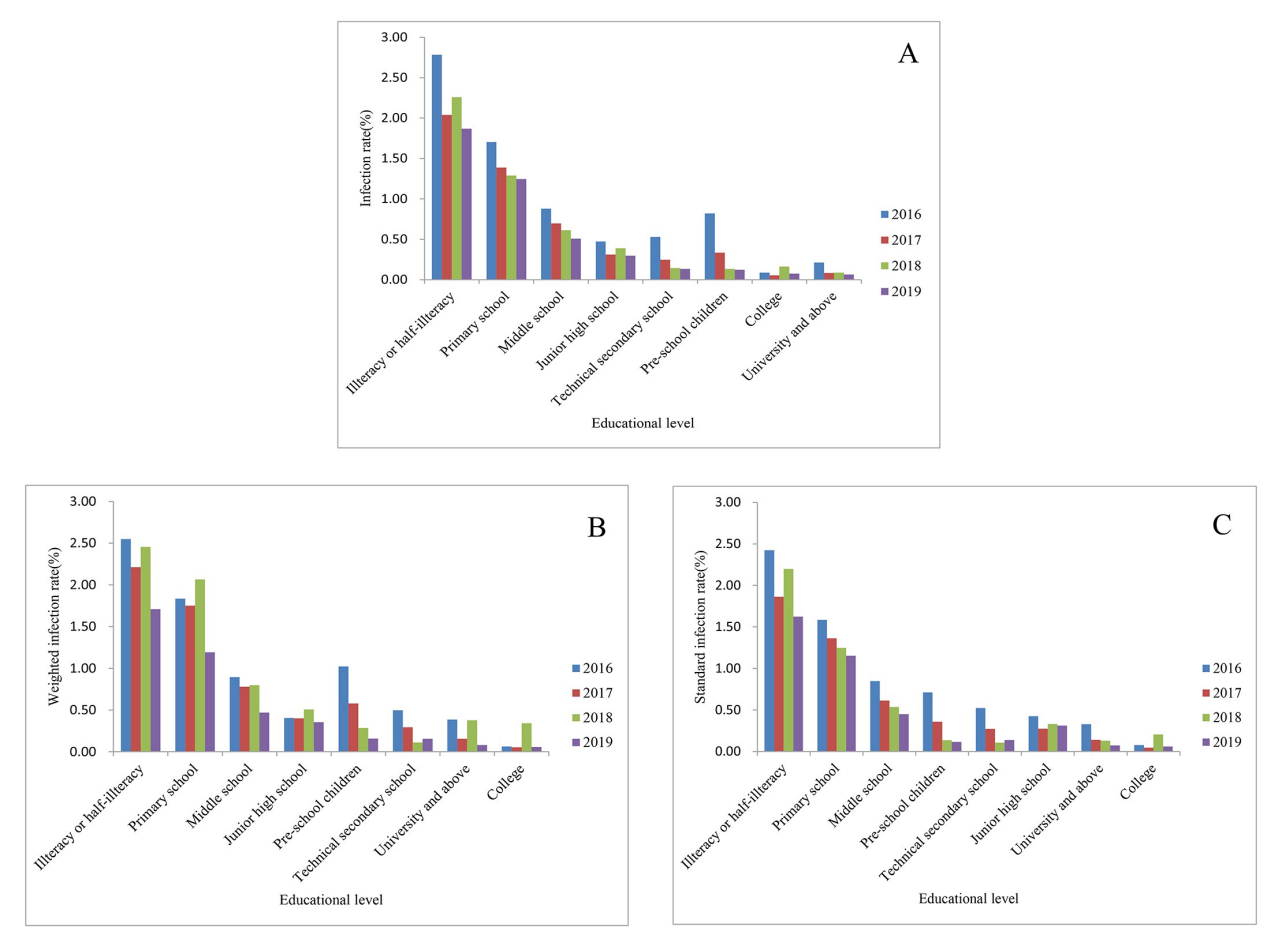
Educational distribution of hookworm infections from 2016 to 2019 according to the national surveillance. A. Infection rate; B. Weighted infection rate; C. Standard infection rate.

**Fig 7:**
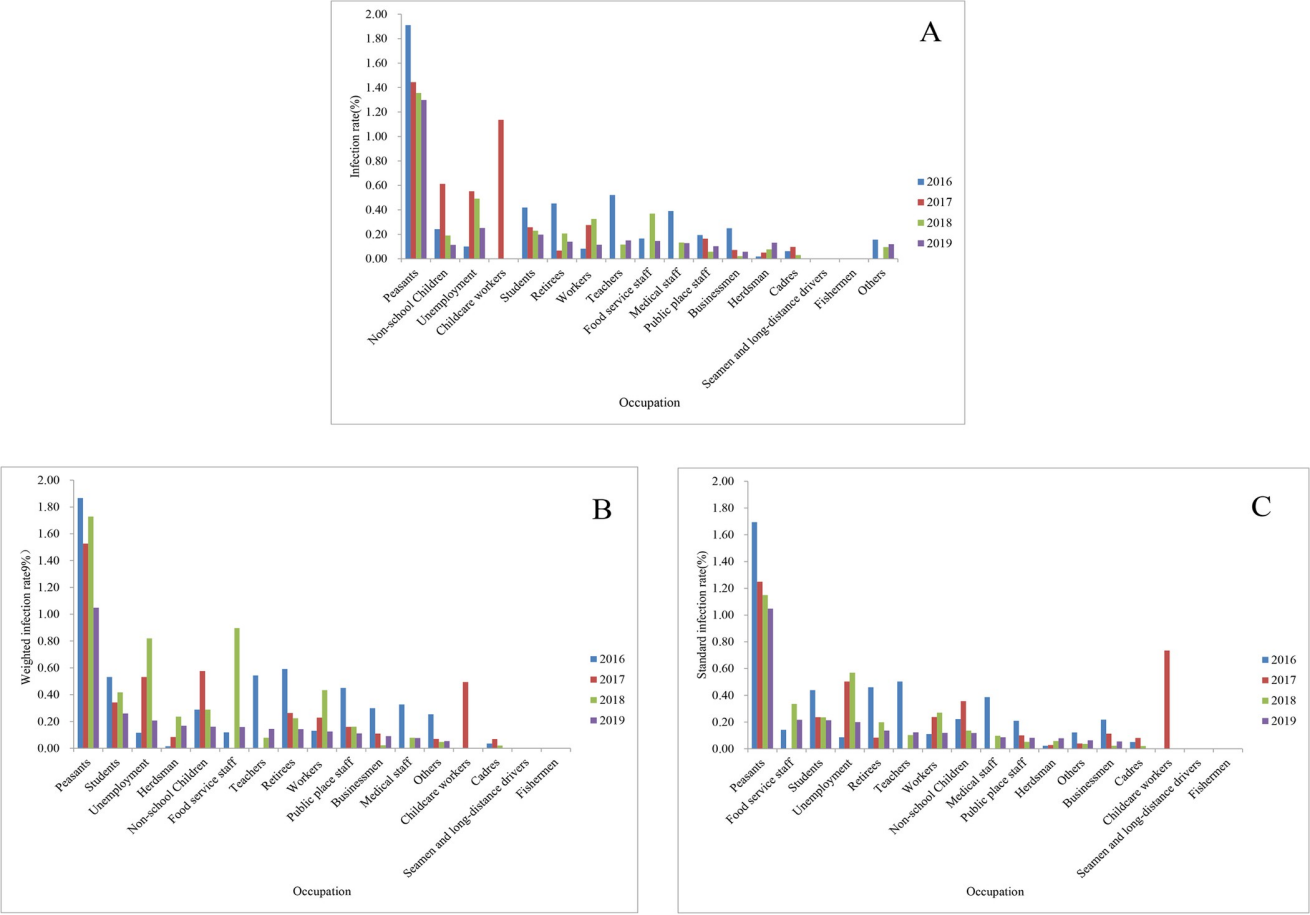
Occupation distribution of hookworm infections from 2016 to 2019 according to the national surveillance. A. Infection rate; B. Weighted infection rate; C. Standard infection rate.

In 2019, the standard infection rate of hookworm in the illiterate or semi-illiterate group, primary school, middle school, university, and college were 1.62% (770551/47452507), 1.15% (2144562/186165439), 0.45% (1052728/234762187), 0.07% (11151/15573639) and 0.06% (12892/21684990), respectively. The standard infection rate of hookworm was the highest among the illiterate or semi-illiterate group, followed by the primary school and middle school from 2016 to 2019. The lowest was the university or college group (Fig 6). The Cochran-Armitage trend test showed a declining trend of infection with increased educational level in all four years’ national surveillance (*P*<0.0001) (Pre-school children not included). In 2019, the highest standard infection rate was among the peasants, which was 1.05% (3864644/368765960), followed by food service staff and students, with the standard infection rate of 0.22% (8546/3921705) and 0.21% (240203/112289079), respectively. Childcare workers, administrative workers, seamen, long-distance drivers, and fishers were not hookworm positive. Moreover, hookworm positive was not found in 2016, 2018, and 2019, except 2 out of 176 positives found in 2017 among childcare workers, with a 1.14% infection rate (Fig 7).

### Infection intensity, discrimination of *N*. *americanus* and *A*. *duodenale*, and soil contamination

**Fig 8:**
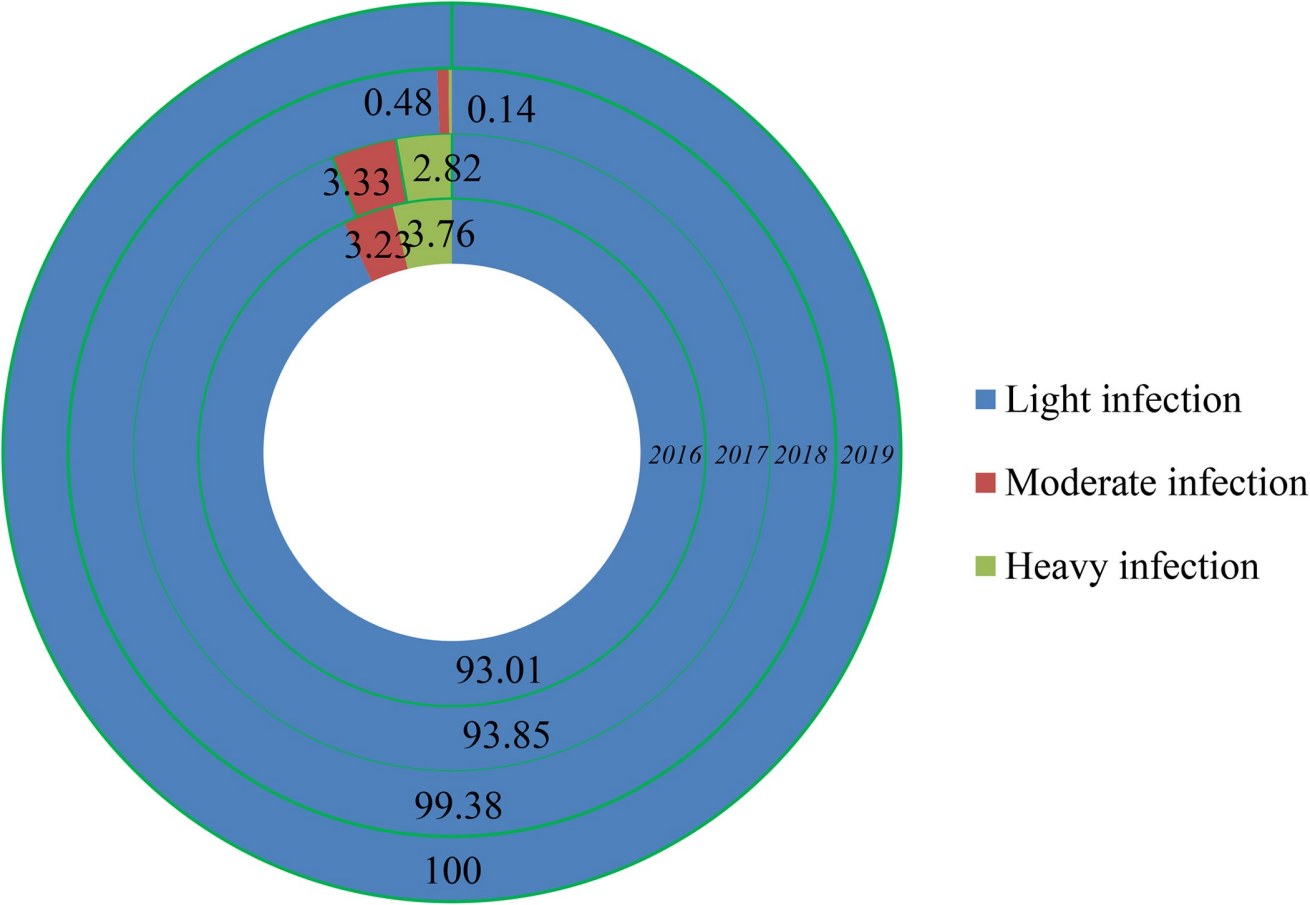
Constitute ratio (%) of infection intensity from 2016 to 2019.

All 3580 positive cases detected were determined as mild infections in 2019. From 2016 to 2019, more than 93% of the cases were mild infections. The ratio of mild infection increased annually, whereas severe infection ratio was decreased (Fig 8). The larva was obtained from 1704 hookworm-infected cases in 2019, and 78.70% (1341/1704) were identified as infected with *N*. *americanus* and 20.31% (346/1704) *A*. *duodenale*. *T*he remaining [1.00% (17/1704)] was infected with both kinds of hookworm.

**Fig 9:**
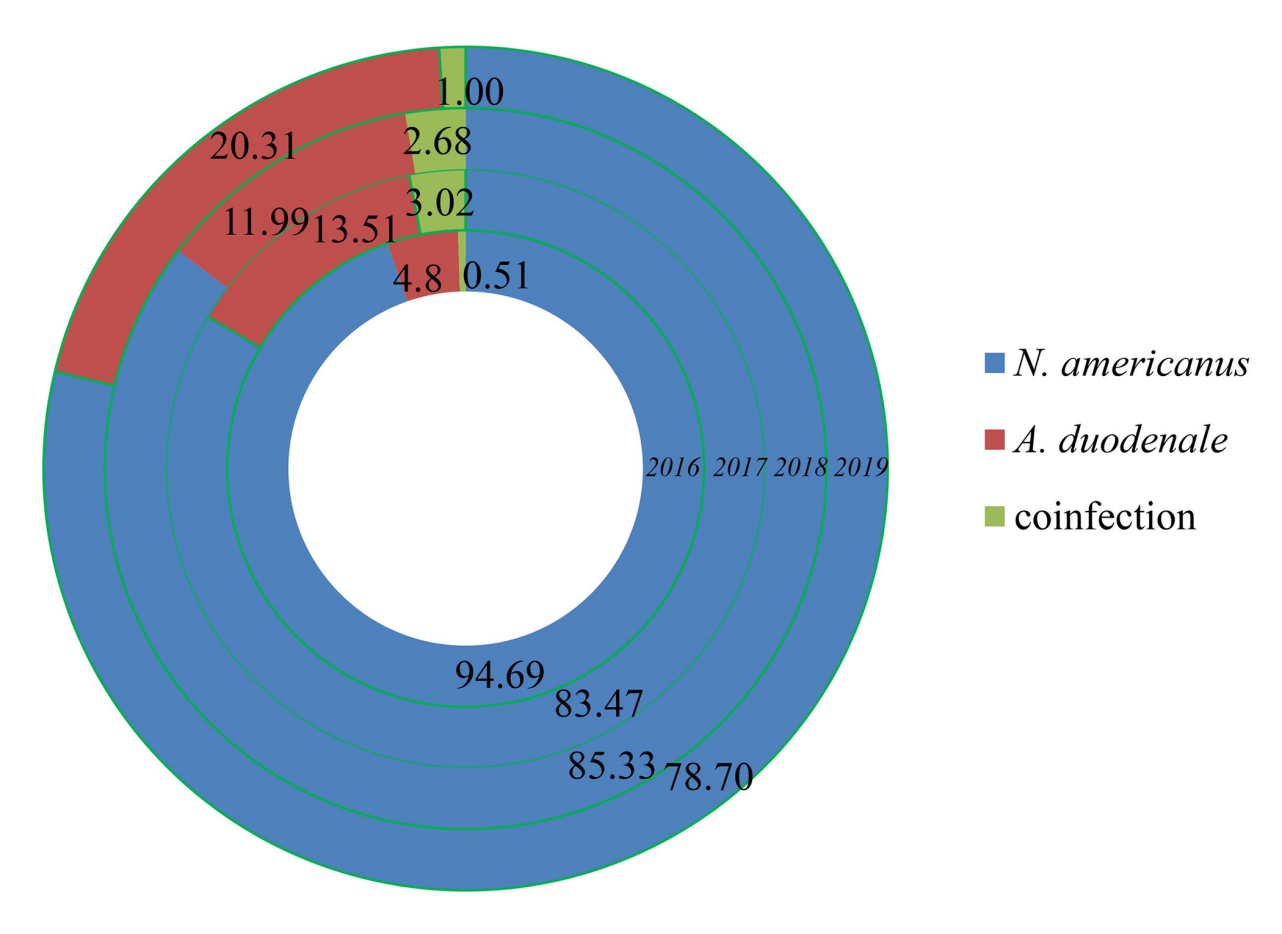
Constituent ratio (%) of hookworm species in China from 2016 to 2019.

From 2016 to 2019, *N*. *americanus* accounts for most hookworm infections, followed by *A*. *duodenale* and coinfections (Fig 9). A total of 2056 soil samples were collected from the households of surveillance spots, and 71 were identified to be contaminated with hookworm larva, with a 3.45% detection rate. The detection rates of hookworm larva in soil from 2016 to 2018 were 2.45%, 3.14%, and 3.45%, respectively.

## Discussion

The national surveillance system has been carried out for 4 years successively and is ideal for understanding hookworm’s endemic status and trends. According to the national surveillances from 2016 to 2019 a declining trend in the overall prevalence of hookworm has been presented from 2016 to 2019. However, the fluctuation was detected in some provinces with high prevalence, such as Yunnan, Hainan, Chongqing, Zhejiang, etc., partly because of the annual changing of surveillance sites and because of the suitable weather for hookworm inhibited with few or no control efforts. These indicate that further control activities should be implemented in these provinces/municipalities. In addition, the hookworm epidemic was characterized by region and population, similar to that of the third national survey [19]. In addition, the infection rate of hookworm is now higher than that of ascariasis and trichuriasis, which was similar to results of the third national survey [19,23–25], indicating the control of hookworm disease has become the priority in controlling soil-transmitted helminthiasis in China.

Hookworm is mainly endemic in the Southern China, mainly in the Southwestern (Tibet not included), Southern, and Southeastern China, especially Sichuan, Yunnan, Chongqing, and Hainan. Conversely, little or no infection was detected in the Northern China, as indicated by the national surveillances for four years from 2016 to 2019 and the third national survey [19,23–25]. The warm and humid weather in the Southern China, together with poverty, poor hygiene conditions, in many villages in the southwestern China favored hookworm transmission. In contrast, the cold and dry weather and the frigid weather that promotes shoes and gloves wearing in the Northern China were not suitable for hookworm. High altitude was the main feature in the Western China, such as Xijiang, Qinghai, Gansu, and Tibet. This area is not ideal for hookworm transmission due to unfavorable climate status, for instance, temperate was relatively low with large temperature difference, rainfall was either too few or too much [28,29]; therefore, few or no hookworm was discovered there. No hookworm was found in 10 P/A/Ms for four successive years which attributed by highly developed economic status in some municipalities such as Beijing, Shanghai, and Tianjin [30] with a good hygienic living environments. However, for Northern provinces, such as Jilin, Liaoning, Heilongjiang, Inner Mongolia, and Qinghai, frigid and dry weather unsuitable for hookworm habitation was the main cause [28,29].

According to results from the 4 years’ national surveillances and the third national survey, the infection rate of hookworm in the females was higher than that of the males. Higher infection rate was mainly distributed in the middle and older age groups; however, it was low in children. This is in consistence with reports of the second and third national survey and investigation abroad [18,19,31]; In villages, the middle and older groups tend to stay more in their home towns and work in the fields while the younger ones work or study in cities. Furthermore, middle-aged and older age groups, especially women, always work in vegetable yards, neither wearing shoes nor gloves, increasing their risk for hookworm infections. Therefore, special attention should be paid to middle age and older women in control hookworm program.

For occupational distribution, the infection rate of peasants was highest among all occupations. No hookworm was found in seamen because opportunities of exposure to the hookworm in seamen are much rarer compare to that of peasants. The hookworm infection rate decreased with increased educational level, highlighting the importance of more educated people to prevent and control hookworm diseases. Subjects with higher educational levels might have better hygiene habits and are less likely to engage in work and other activities that lead to exposure to infection. In addition, they tend to be more informed about hookworm disease through books, mass media, and other channels.

Compared with *A*. *duodenale*, *N*. *americanus* still accounted for most of the hookworm infections in China in 2019 that is in consistence with results from the third national survey and the national surveillance from 2016 to 2018 [19,23–25]. According to the first national survey, the ratio of *A*. *duodenale* to *N*. *americanus* was 1.017:1, and they were mainly distributed in the Northern and Southern China, respectively. The lower prevalence of *N*. *americanus* was partly due to its limited distribution and less transmissible compare to that of *A. duodenale*. The two benzimidazole drugs, e.g. mebendazole and albendazole, have been widely used against human infections in China, and albendazole appeared to be more effective than mebendazole in hookworm infections [32–39]. Moreover, the efficacy of albendazole in *A*. *duodenale* infections was better than for *N*. *americanus* [40], indicating development of more efficient drugs to cure *N. americanus* is necessary to reduce its infection rate in the future.

High and moderate infection was found in surveillance before 2019 and the third national survey; however, no heavy infection was observed in 2019. Lower infection intensity account for less egg discharged into the environment so that hookworm disease transmission was inhibited. In contrast, lower infection intensity will make the microscope examination a more difficult task [23]. The Kato-Katz method is the most widely used method due to its simplicity and low cost [41]. It is recommended by the WHO for quantifying STH eggs in the human stool [42]. According to a meta-analysis comparing the sensitivities of the most commonly used copro-microscopic diagnostic methods for STH, namely Kato-Katz, direct microscopy, formol-ether concentration, McMaster, FLOTAC, and Mini-FLOTAC the highest sensitivity was observed for the FLOTAC method, whereas the sensitivity of the Mini-FLOTAC method was comparable with the Kato-Katz method. To detect low-intensity hookworm, the sensitivities of one-slide Kato-Katz, two-slide Kato-Katz, two-sample Kato-Katz and three-sample Kato-Katz were 41.2%, 52.6%, 55.8% and 56.1%, respectively, and the sensitivities of FLOTAC and Mini-FLOTAC were 68.8% and 44.3%, separately [43]. The FLOTAC method has relatively higher sensitivity; however, it requires large equipment, such as centrifuge [44,45], making it difficult to be generalized in the field investigation. And in the condition of low infection intensity using Kato-Katz method, an increasing number of stool samples will only result in a slight increment in sensitivity. However, the cost of human resources, materials, and financials would have to be greatly increased in such large-scale field investigations. Under overall consideration, the Kato-Katz method (one sample for two slides) was commonly used in the national surveillance, but there are limitation in sensitivy and the infection rate of hookworm might be underestimated, which is a serious question needs to be taken into consideration in future surveillance.

## Conclusion

China has made significant progress in controlling hookworm disease and is currently at the stage of interruption and elimination. The upcoming national control program in 2021–2025 has set the goals and will provide support for hookworm disease control further. Many aspects should be considered to achieve these goals. Special attention should be paid to the high endemic areas by considering the uneven endemic status of hookworm infections. Precision control measures should be implemented according to the local settings. Further, the national surveillance system should be continually strengthened to provide necessary information for control programs.
